# Radiomics-Based Machine Learning Models for Predicting P504s/P63 Immunohistochemical Expression: A Noninvasive Diagnostic Tool for Prostate Cancer

**DOI:** 10.3389/fonc.2022.911426

**Published:** 2022-06-20

**Authors:** Yun-Fan Liu, Xin Shu, Xiao-Feng Qiao, Guang-Yong Ai, Li Liu, Jun Liao, Shuang Qian, Xiao-Jing He

**Affiliations:** ^1^ Department of Radiology, The Second Affiliated Hospital of Chongqing Medical University, Chongqing, China; ^2^ Big Data and Software Engineering College, Chongqing University, Chongqing, China

**Keywords:** P504s/P63, machine learning, MRI, immunohistochemistry, prostate cancer

## Abstract

**Objective:**

To develop and validate a noninvasive radiomic-based machine learning (ML) model to identify P504s/P63 status and further achieve the diagnosis of prostate cancer (PCa).

**Methods:**

A retrospective dataset of patients with preoperative prostate MRI examination and P504s/P63 pathological immunohistochemical results between June 2016 and February 2021 was conducted. As indicated by P504s/P63 expression, the patients were divided into label 0 (atypical prostatic hyperplasia), label 1 (benign prostatic hyperplasia, BPH) and label 2 (PCa) groups. This study employed T2WI, DWI and ADC sequences to assess prostate diseases and manually segmented regions of interest (ROIs) with Artificial Intelligence Kit software for radiomics feature acquisition. Feature dimensionality reduction and selection were performed by using a mutual information algorithm. Based on screened features, P504s/P63 prediction models were established by random forest (RF), gradient boosting decision tree (GBDT), logistic regression (LR), adaptive boosting (AdaBoost) and k-nearest neighbor (KNN) algorithms. The performance was evaluated by the area under the ROC curve (AUC) and accuracy.

**Results:**

A total of 315 patients were enrolled. Among the 851 radiomic features, the 32 top features were derived from T2WI, in which the gray-level run length matrix (GLRLM) and gray-level cooccurrence matrix (GLCM) features accounted for the largest proportion. Among the five models, the RF algorithm performed best in general evaluations (microaverage AUC=0.920, macroaverage AUC=0.870) and provided the most accurate result in further sublabel prediction (the accuracies of label 0, 1, and 2 were 0.831, 0.831, and 0.932, respectively). In comparative sequence analyses, T2WI was the best single-sequence candidate (microaverage AUC=0.94 and macroaverage AUC=0.78). The merged datasets of T2WI, DWI, and ADC yielded optimal AUCs (microaverage AUC=0.930 and macroaverage AUC=0.900).

**Conclusions:**

The radiomic-based RF classifier has the potential to be used to evaluate the presurgical P504s/P63 status and further diagnose PCa noninvasively and accurately.

## 1 Introduction

Prostate cancer (prostate carcinoma, PCa) is the most prevalent non-cutaneous malignancy among males worldwide, with approximately 1.4 million new diagnosed cases according to GLOBALCAN 2020 (7.3% of all new cancer cases) (1). In 2021, an estimated 248,530 new cases of PCa and 34,130 deaths occurred in the USA alone (2). Moreover, one study reported an excess of 8.4 billion euros (7% of overall cancer costs) related to the disease (3), imposing immense health and economic burdens worldwide.

Protein-specific antigen (PSA) screening has traditionally been the main PCa screening approach and has facilitated the early detection of PCa in recent decades. Nevertheless, benign prostatic hyperplasia (BPH), prostatitis and other noncancerous diseases may also cause an elevate in PSA serum levels. A large percentage of false-positive results of PSA, leading to unnecessary biopsies and healthcare costs, have plagued both clinicians and suspected PCa patients (4).

Till such time, the golden standard of PCa diagnosis remains a pathological one. However, it’s tough for pathologists to make a definitive diagnosis just based on nucleus and cytoplasm morphological discrepancies, especially in some ambiguous cases. In surgical pathology practices, immunohistochemistry (IHC) staining has long been acknowledged as an essential tool for assisting in the correct diagnosis and subclassification of malignant neoplasms, which provided much information about diseases such as immune microenvironment and dysregulated gene products at the sub-cellular level. In recent years, IHC biomarkers, especially P63 and alpha-methyl acyl-CoA racemase (AMACR, P504s), have attracted growing attention and been used widely in clinical practice. PCa shows the absence of the basal cell layer compared to benign prostate tissues. While P63, expressed by the basal cells, could be regarded as a kind of negative marker of PCa. On the other hand, P504s, an enzyme involved in fatty acid metabolism, have been proved expression upregulated in 97–100% of PCa (5, 6). Therefore, P504s can function as a positive biomarker for diagnosis of prostate cancer. The combined application of P504s and P63 have efficiently promoted advances in prostatic disease diagnosis. The International Society of Urologic Pathology (ISUP) recommended utilizing a double cocktail combining P504s and P63 for the workup of “suspicious prostatic adenocarcinoma foci” as well (7). Patients would be classified into 4 different diagnosis categories according to the staining results: 1) benign lesion (P504s negative/P63 positive); 2) prostate cancer (P504s positive/P63 negative); 3) high-grade prostatic intraepithelial (HGPIN) or atypical adenomatous hyperplasia (AAH, P504s positive/P63 positive); and 4) atypical small acinar proliferation, suspected to be malignant (ASAP, P504s negative/P63 negative) (8).

However, it is invasive to obtain histopathological specimens for detection of P504s/P63 through biopsy or transurethral resection of the prostate (TURP). And patients would experience different degrees of anxiety and complications such as bleeding, infection and erectile dysfunction (9, 10). Although there were some efforts to eliminate these influence, for example, G.M. Busetto et al. demonstrated a dual 5α-reductase inhibitor could help decrease TURP bleeding loss in large prostate (>50ml) (11), the underlying concerns had not been fully resolved. Development of a new and precise but noninvasive method for predicting immunohistochemical results is necessary.

Magnetic resonance imaging (MRI) has become an integral part of PCa diagnostic procedures due to its satisfactory soft tissue contrast and multidimensional information. The most recent PCa guidelines (12) recommended utilizing routine prebiopsy multiparametric MRI (mpMRI) to localize suspicious targeted areas. However, subjective elements exist in MRI experts’ visual assessments despite the newly published Prostate Imaging Reporting and Data System version 2.1 (PI-RADS V2.1) (13) promoting a more standardized reporting quality. Moreover, the diagnostic performance of MRI does not remain at the pathological diagnosis level. Delightfully, advances in radiomics and machine learning (ML) technologies offer potentially promising solutions (14), which are expected to objectively and noninvasively predict immunohistochemical results of pathology from image-extracted features. In fact, some reports of radiomics have indeed predicted molecular profiles, which are clinically important. For instance, Chad Tang et al. (15) first predicted non-small-cell lung cancer prognoses *via* a radiological model of immunopathological information based on the relationship between the tumor immune microenvironment and survival. Li Jing et al. (16) also reported that high-order radiological features based on T2 fluid-attenuated inversion/recovery (FLAIR) MRI used to predict IHC glioma features could provide personalized treatment guidance for patients. In recent years, some studies have demonstrated that MRI parameters such as the apparent diffusion coefficient (ADC) have strong correlations with the expression of P504s, Ki-67 and HIF-1α in PCa patients. In addition, Shukla-Dave et al. found that MRI combined with molecular profiles such as Ki-67 provided incremental value to clinical variables regarding PCa recurrence prediction (17–19). Nonetheless, no ML-related studies have yet been reported that predict prostate IHC markers such as P504s and P63 expression.

We hypothesized that the signal intensity differences in MRI might reflect the subtle changes in microstructures in histopathology. Exploring the potential predictive value of MRI radiomics in pathological indices could contribute to the interpretability of ML models, improve MRI analyses, and reveal the connections between tumor microenvironment changes and MRI imaging. Thus, based on MR radiomic features, we constructed ternary classification models with five different ML techniques to identify the P504s/P63 status to further achieve the goal of noninvasive and accurate PCa diagnosis.

## 2 Materials and Methods

### 2.1 Population

**Figure 1:**
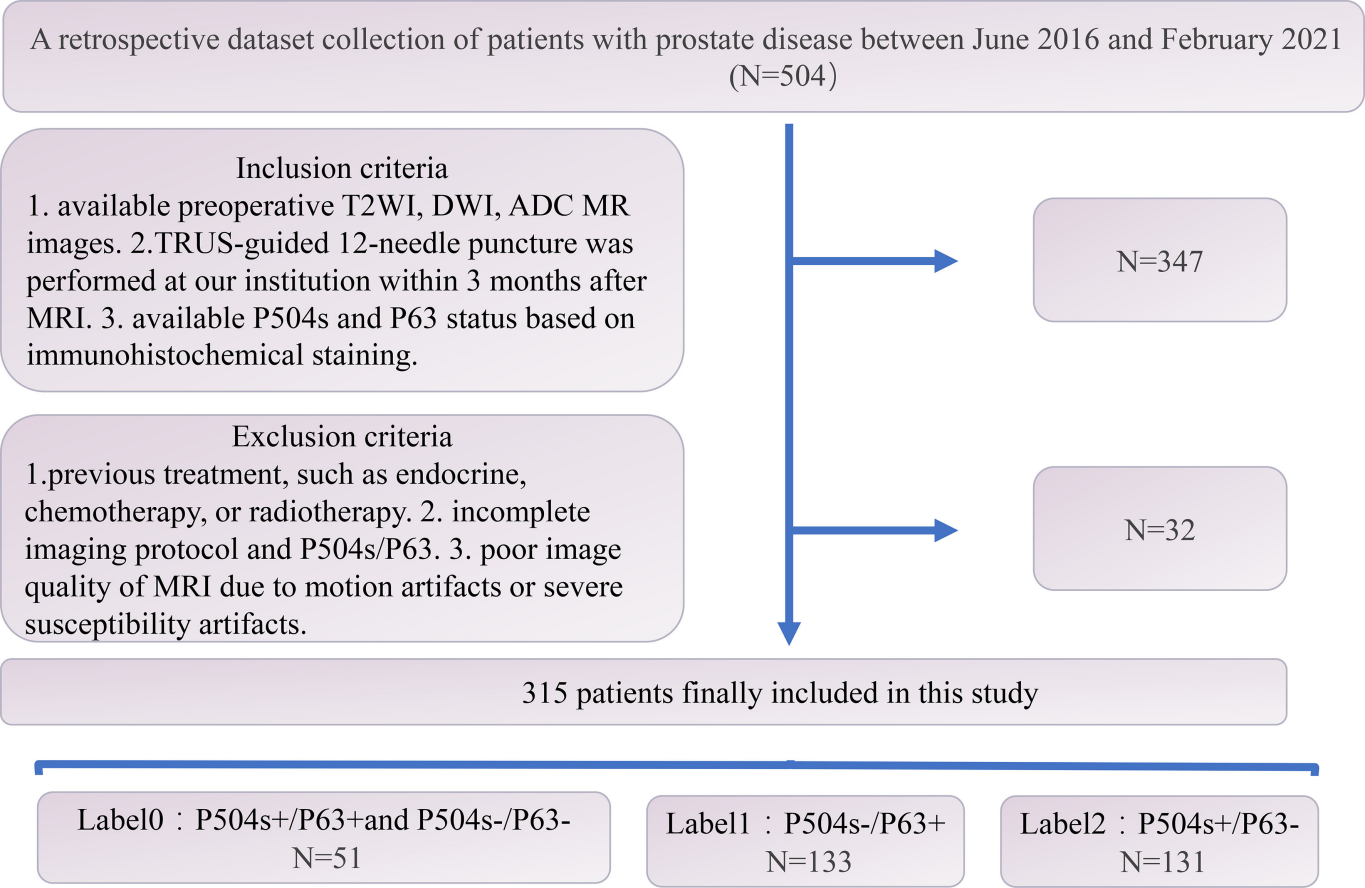
Flow chart of the patient selection process.

The institutional review board of our hospital approved this retrospective study and waived the requirement for informed consent (decision number (2019) 289). Patients who underwent prostate MRI scanning between June 2016 and February 2021 were screened for inclusion. The specific inclusion and exclusion criteria are described in **Figure 1**. Of the 347 eligible patients, 32 were excluded based on the exclusion criteria, leaving 315 patients involved in this study.

### 2.2 MRI Protocol

MR images were acquired by using a 3.0-T MRI scanner (MAGNETON Prisma; SIEMENS A Tim Dot System) and an 8-channel phased-array software coil. The scan covered the prostate gland, seminal vesicle glands and as many adjoining structures as possible. The pertinent sequences for the study were the axial fat suppression (FS) T2-weighted imaging (T2WI) sequences with the following imaging parameters: repetition time (TR): 3090 ms; echo time (TE): 77 ms; slice thickness: 3 mm; number of excitations (NEX): 2; field of view (FOV): 20×20 cm; and acquisition matrix: 320 ×240. Diffusion-weighted imaging (DWI) sequences were obtained in the axial plane, where the orientation and location were identical to those prescribed for axial FS T2-weighted MRI, and the data were obtained by using axial echoplanar imaging (EPI) sequences as follows: TR: 3800 ms; TE: 84 ms; slice thickness: 3 mm; NEX: 2; FOV: 20×20 cm; acquisition matrix: 118 ×118; and b values: 1400 s/mm2. ADC maps were generated by using a designated workstation (Advanced Workstation 4.6; GE Medical Systems; FUNCTOOL).

### 2.3 IHC Status

All patients underwent a transrectal ultrasound (TRUS)-guided 12-core systematic biopsy performed by experienced urologists within 3 months after the MRI examinations. Biopsy tissue cores were individually labeled according to their locations and analyzed by an experienced uropathologist. IHC staining was performed with the following antibodies: anti-P63 (rat monoclonal [MAB-0694], MX013, Maixin) and anti-P504s (rabbit monoclonal [RMA-0546], 13H4, Maixin). The same genitourinary pathologist assessed P63-positive expression based on the linear and continuous nuclear staining of basal cells and P504s-positive expression based on the uninterrupted, dark, cytoplasmic or apical granular staining of prostate epithelial cells (20). Patients were classified into 3 groups according to their P504s/P63 staining results as described below: label 0: both high-grade PIN or AAH (P504s positive/P63 positive) and ASAP (P504s negative/P63 negative); label 1: benign lesion (P504s negative/P63 positive); and label 2: prostate cancer (P504s positive/P63 negative).

### 2.4 Radiomic Feature Extraction

**Figure 2:**
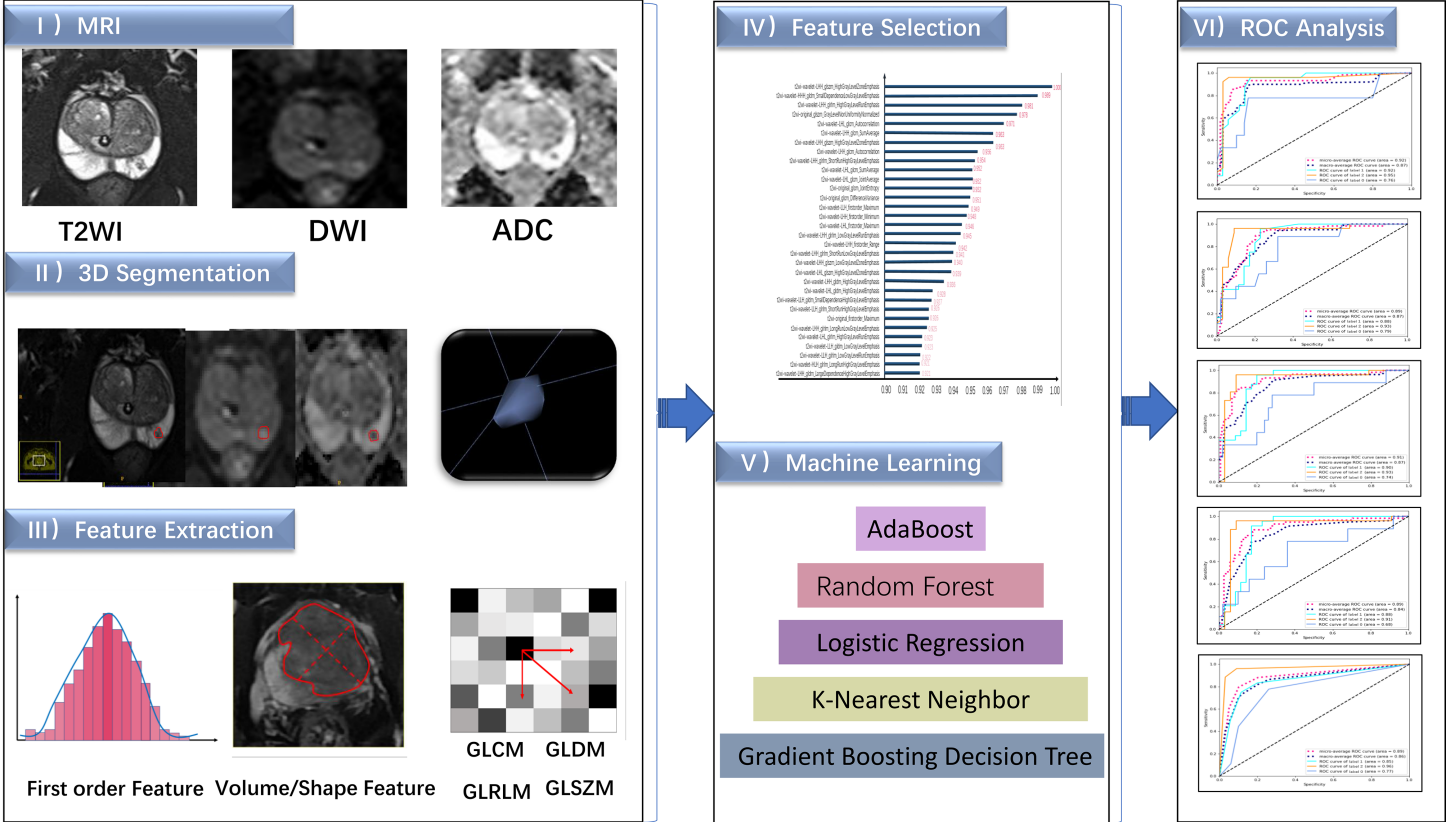
The flow chart of this study.

Two radiologists who have worked in abdominal imaging for more than 10 years reviewed the MRI results, including the T2WI, DWI and ADC images. Hyperintense signal regions on DWI (b value = 1400) and hypointense signal regions on T2WI and ADC were regarded as tumor areas. In cases of multifocal prostate cancer, only the largest lesion was analyzed. All disagreements were discussed until a consensus was reached. Subsequently, the radiologists finished the three-dimensional segmentation. Feature extraction processes for the benign prostatic hyperplasia (BPH) transitional zone and suspicious PCa areas were performed with AK software (Artificial Intelligence Kit, GE Healthcare). A total of 107 features were obtained from a single sequence of each patient: 18 based on first-order statistics, 14 based on 3D shapes, and 75 based on textures. The texture features were further subdivided into 5 categories: 24 belonged to the gray-level cooccurrence matrix (GLCM), 16 belonged to the gray-level run length matrix (GLRLM), 16 belonged to the gray-level size zone matrix (GLSZM), 14 belonged to the neighboring gray tone difference matrix (NGTDM), and 5 belonged to the gray-level dependence matrix (GLDM). Moreover, considering that a wavelet provides spatial and frequency representations of the corresponding signal, features preprocessed with the wavelet filter were also extracted from the images. Hence, a total of 851 features were extracted from the T2WI, DWI and ADC images of each patient.The patients were split into a training set (n = 252 patients) and a testing set (n= 59 patients). Feature selection and dimensionality reduction were performed exclusively on the training set. The entire technical flow chart of this study is depicted in **Figure 2**.

### 2.5 Construction of Immune Radiomic-Based Diagnostic ML Models

In this study, we integrated the radiomic features retained from the T2WI, DWI, and ADC images into composite diagnostic models for P504s/P63 prediction *via* immune signatures by applying ML algorithms in the derivation cohort. Five ML algorithms, namely, 1) random forest (RF), 2) logistic regression (LR), 3) gradient boosting decision tree (GBDT), 4) k-nearest neighbor (KNN), and 5) adaptive boosting (AdaBoost) algorithms, were independently applied to select and combine multiple covariates from the candidate features. An algorithm called k-means clustering with the synthetic minority oversampling technique (k-Means-SMOTE), was used to reduce noise and further boost the performance of the classifiers. The final optimal models were then trained on the selected covariates and the optimized algorithm parameters.

### 2.6 Statistical Analysis

Statistical analysis software (SPSS, version 25; IBM Corporation, Armonk, NY, USA) was used for patient characteristics data analysis with p-value < 0.05 indicating statistical significance. The distributions of continuous variables were checked for normality *via* the Shapiro-Wilk test. Normally distributed variables are shown as the mean ± standard deviation (SD), and nonnormally distributed variables are expressed as the medians with interquartile ranges in parentheses (25th and 75th percentiles). Variance analyses and homogeneity comparisons among the groups were made with one-way analysis of variance (ANOVA).

The radiomic features and models’ performance statistical analysis was implemented by using the Pycharm platform and Python SCIKIT-LEARN (version 0.18.1). The mutual information (MI) feature selection algorithm was used to identify and rank order the top 32 radiomic features that distinguish three labels within the training set. The mutual information calculation formula is as follows:


$$I\left(X;Y\right)=\sum_{x\in X}\sum_{y\in Y}P\left(x,y\right)log\frac{P\left(x,y\right)}{P\left(x\right)P\left(y\right)}$$

In this study, X represents the selected radiological characteristics, and Y represents the final characteristic coefficient.

To ensure generalizability, the five ML models were optimized through five times repeated 5-fold cross-validation. Algorithms performance was assessed using the receiver operating characteristic (ROC) curves and areas under the ROC curves (AUCs). And further analyses included contingency tables for assessing sensitivity, specificity, the Youden index, positive and negative predictive values (PPVs and NPVs, respectively), and Brier scores.

## 3 Results

### 3.1 Patient Characteristics

A total of 315 patients (average age: 70.56 ± 8.337; range: 42~89) were involved in this study, among which 51 were classified as 0 (average age: 70.784 ± 7.412), 133 as label 1 (average age: 69.421 ± 8.793) and 131 as label 2 (average age: 71.626 ± 8.108). The three groups showed no significant differences in age (F=2.351; P=0.097> 0.05).

### 3.2 Feature Extraction and Selection

**Figure 3:**
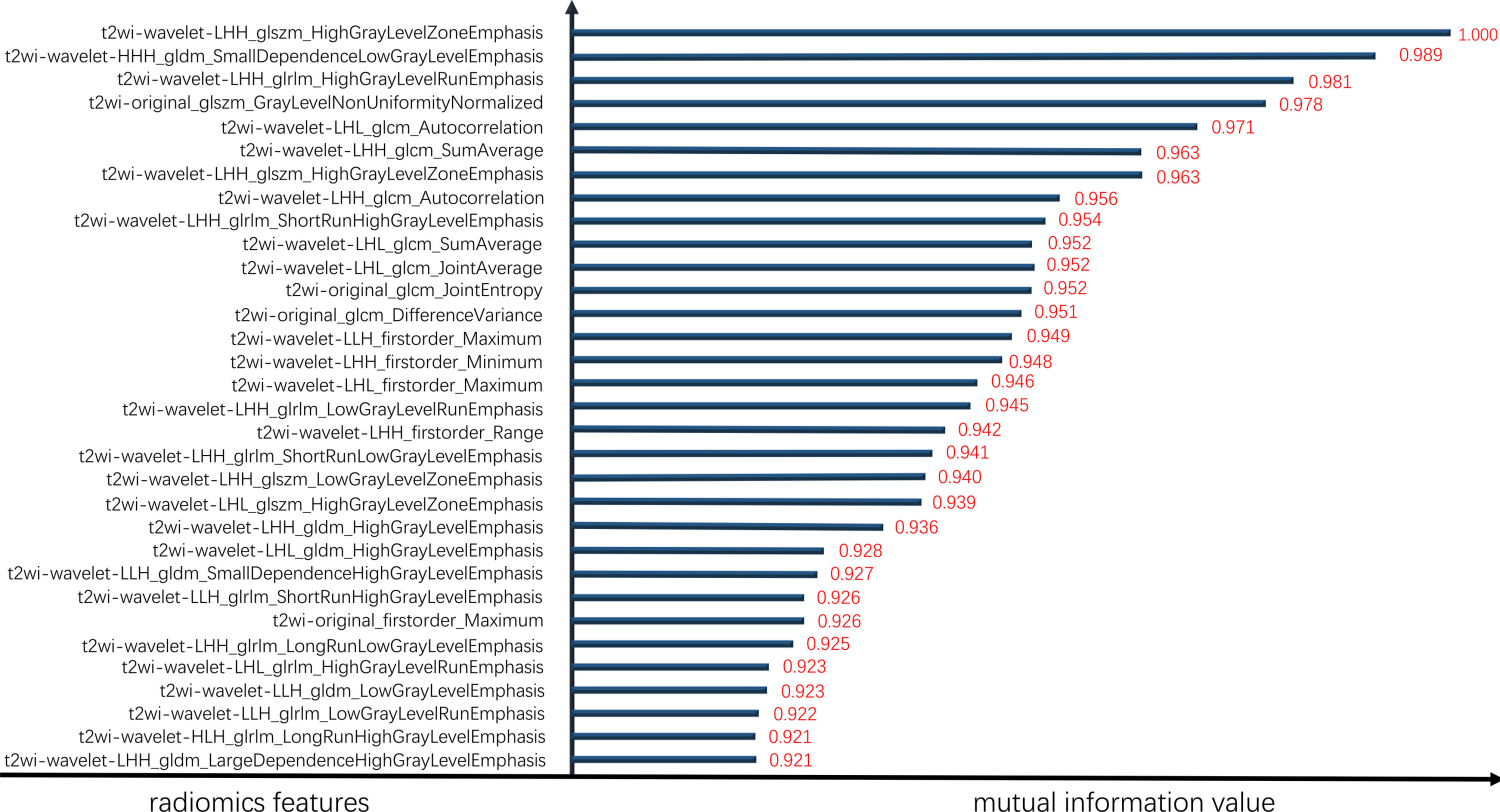
Sorted element mutual information values.

Features were screened from the merged dataset (comprising T2WI, DWI and ADC images) according to the mutual information method. A total of 32 modeling features derived from the T2WI images were finally adopted, of which 4 were original features and 28 were wavelet features. Twenty-seven texture features (9 GLRLM, 7 GLCM, 6 GLDM and 5 GLSZM) and five first-order statistics were obtained. The metrics for the features of the merged dataset model are summarized in **Figure 3**. The retained features are listed in descending order of their variable contributions.

### 3.3 ML Models for P504s/P63 Prediction

#### 3.3.1 ML Algorithm Comparison

**Figure 4:**
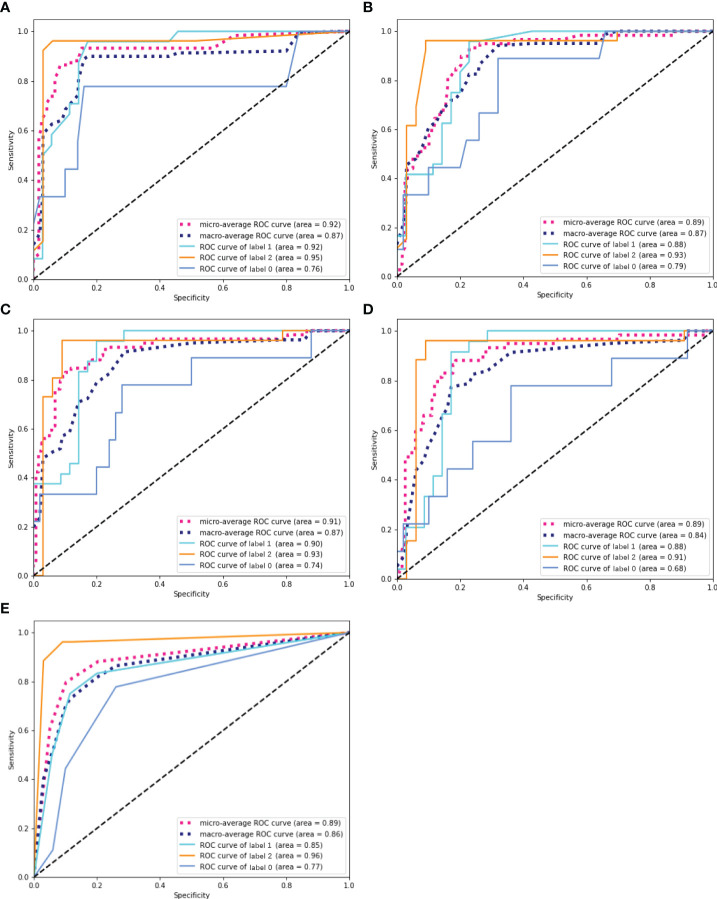
ROC curves of five ML prediction models. **(A)** ROC curve of RF. **(B)** ROC curve of AdaBoost. **(C)** ROC curve of GBDT. **(D)** ROC curve of LR. **(E)** ROC curve of KNN. The solid line represents the prediction efficiency of each group in the corresponding model. Dotted lines represent the overall prediction performance of the models, including the macro-average ROC curves and micro-average ROC curves.

The overall prediction effectiveness of the five models is displayed in **Figure 4**, and the RF algorithm yielded a better AUC (microaverage AUC=0.920, macroaverage AUC=0.870) than the GBDT(microaverage AUC=0.910, macroaverage AUC=0.870), LR(microaverage AUC=0.890, macroaverage AUC=0.840), AdaBoost(microaverage AUC=0.890, macroaverage AUC=0.870) and KNN algorithms (micro-average AUC=0.890, macro-average AUC=0.860). As summarized in **Table 1**, the RF algorithm also obtained the best overall estimated values (the recall, F1 score and accuracy of the RF were 0.740, 0.750 and 0.850, respectively).

**Table 1:** Results obtained by different ML algorithms.

Models	AUC		Brier-score	Precision	Recall	F1-score	Accuracy
	Micro-average	Macro-average					
RF	0.920	0.870	0.407	0.820	0.740	0.750	0.850
GBDT	0.910	0.870	0.441	0.870	0.690	0.690	0.800
LR	0.890	0.840	0.746	0.640	0.640	0.640	0.760
AdaBoost	0.890	0.870	0.610	0.750	0.700	0.700	0.800
KNN	0.890	0.860	0.610	0.720	0.720	0.720	0.690

AUC, area under the curve; RF, random forest; GBDT, gradient boosting decision tree; LR, logistic regression; KNN, k-nearest neighbours.

We also conducted sublabel prediction performance analyses on the different algorithms. **Table 2** illustrates the forecasting indices of the five models, and all the models achieved satisfactory label classification performance (the accuracies of label 0, 1, and 2 were 0.831, 0.831, and 0.932, respectively).

**Table 2:** Interlabel predictive performance of five ML models.

Models	label	Sensitivity	Specificity	PPV	NPV	Youden index	Accuracy
RF	0	0.333	0.980	0.750	0.891	0.313	0.831
	1	0.917	0.857	0.815	0.938	0.774	0.831
	2	0.962	0.909	0.893	0.968	0.871	0.932
GBDT	0	0.222	1.000	1.000	0.877	0.222	0.881
	1	0.875	0.829	0.778	0.906	0.704	0.847
	2	0.962	0.848	0.833	0.966	0.810	0.898
LR	0	0.222	0.900	0.286	0.865	0.122	0.797
	1	0.750	0.829	0.750	0.829	0.579	0.797
	2	0.961	0.909	0.893	0.968	0.871	0.932
AdaBoost	0	0.333	0.920	0.429	0.885	0.253	0.831
	1	0.792	0.857	0.792	0.857	0.649	0.831
	2	0.962	0.909	0.893	0.968	0.871	0.932
KNN	0	0.444	0.900	0.444	0.900	0.334	0.831
	1	0.750	0.886	0.818	0.838	0.636	0.831
	2	0.962	0.909	0.893	0.968	0.871	0.932

AUC, area under the curve; PPV, positive predictive value; NPV, negative predictive value; RF, random forest; GBDT, gradient boosting decision tree; LR, logistic regression; KNN, k-nearest neighbours.

**Figure 5:**
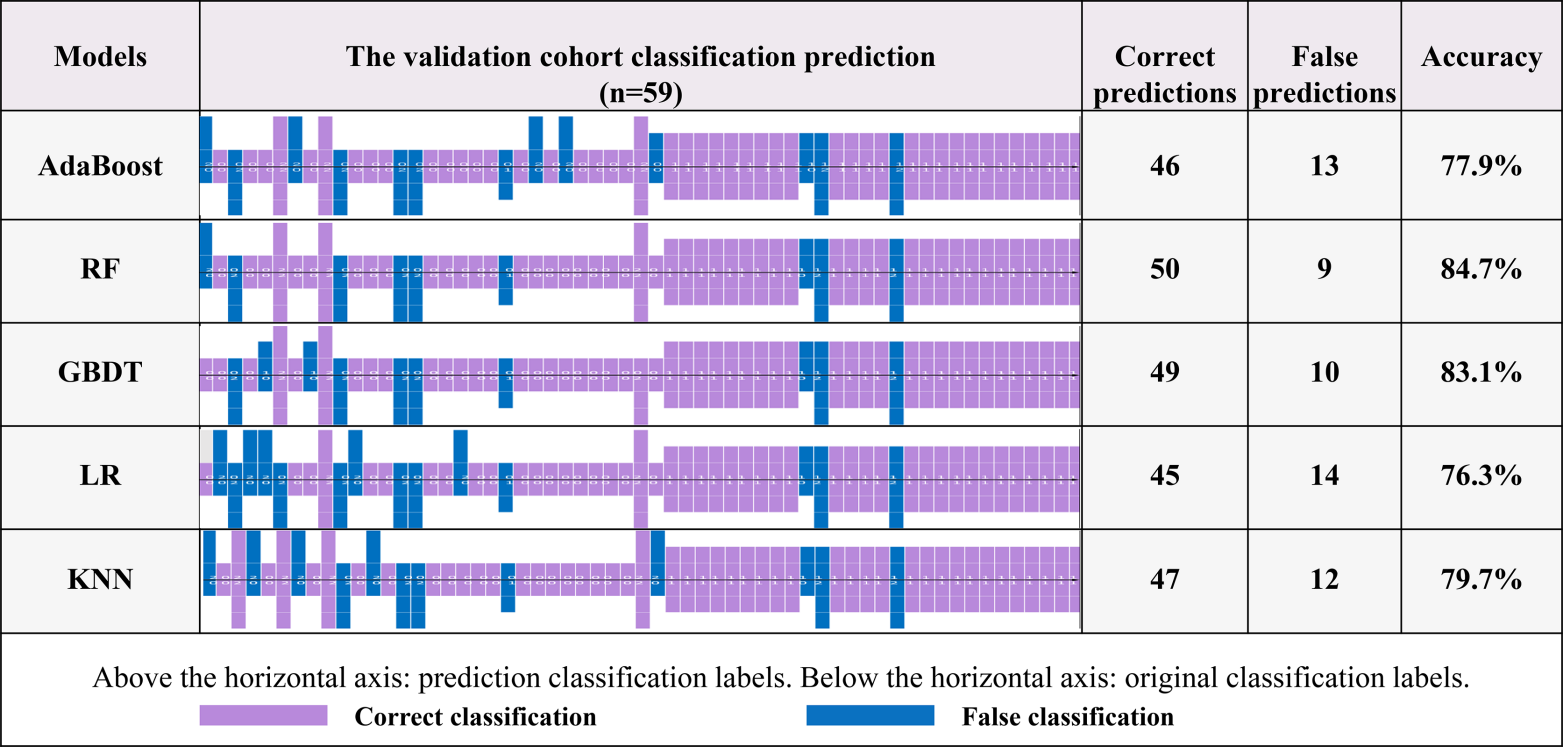
The recognition effects of five prediction models. The information above the horizontal axis represents the model prediction grouping, and that below this axis represents the actual grouping. Purple represents the correct prediction group, and blue represents a case of misrecognition by the corresponding model.

As shown in **Figure 5**, additional verification analyses were performed on an independent validation cohort (N=59), and the results showed that the highest accuracy was achieved by the RF algorithm, which was consistent with the performance on the training cohort (the accuracies of the validation cohort and training cohort were 84.7% and 85.0%, respectively).

#### 3.3.2 Comparative Sequence Datasets

**Figure 6:**
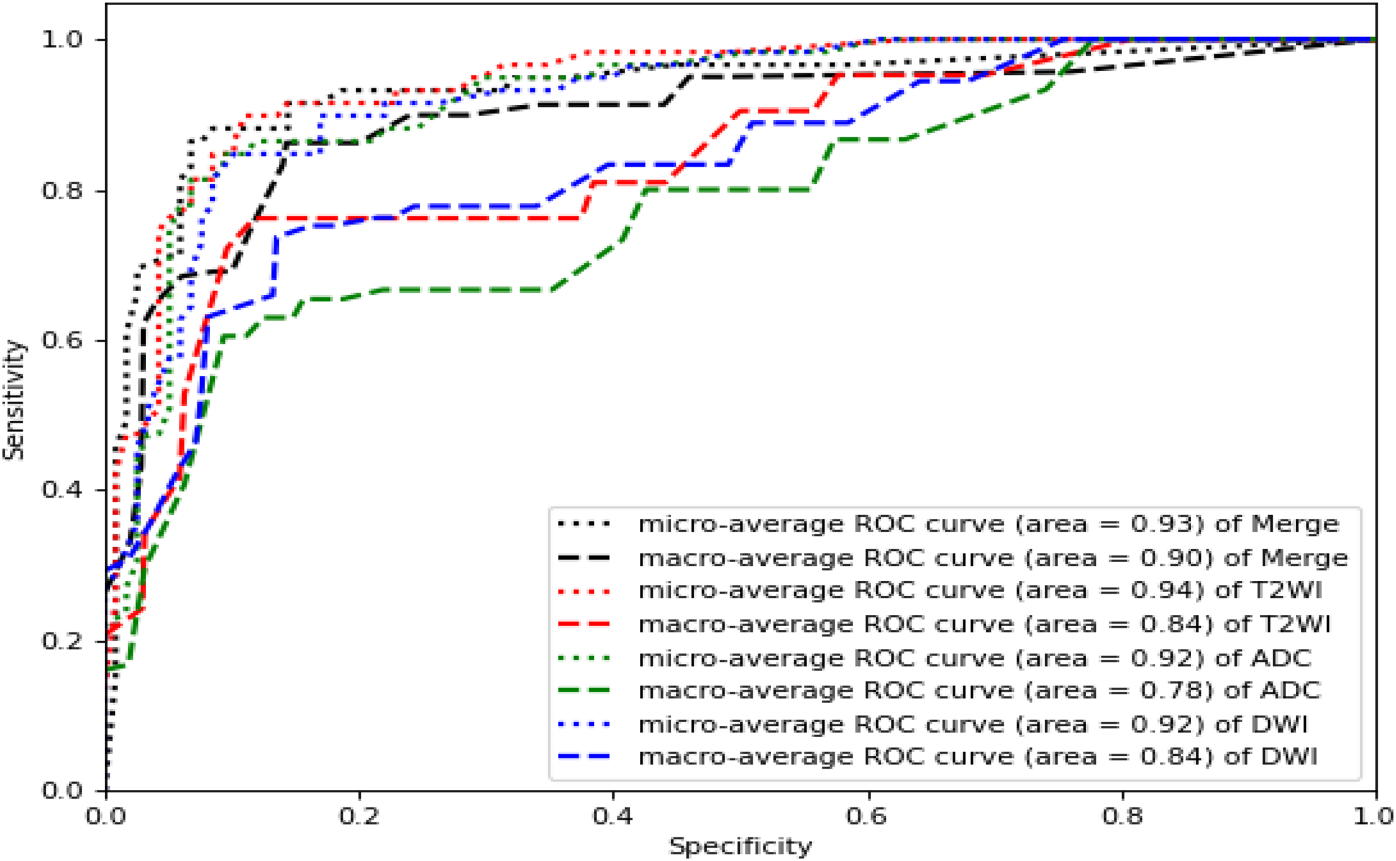
ROC curves of the RF models established by T2WI (red), DWI (blue), ADC (green) and merged sequences (black). The corresponding areas under the micro-average ROC curves were 0.94, 0.92, 0.92, and 0.93, respectively. The corresponding areas under the macro-average ROC curves were 0.78, 0.84, 0.84 and 0.90, respectively.

Based on the best model RF mentioned above, we compared the performance of the single T2WI, DWI and ADC sequence features with that of the merged datasets. As shown in **Figure 6**, T2WI was the best-performing candidate among the single sequences (microaverage AUC=0.94; macroaverage AUC=0.78), but T2WI, DWI and ADC worked best under the merged condition (microaverage AUC=0.930; macroaverage AUC=0.900, accuracy=0.850). The numerical results representing the effects of different datasets are summarized in **Table 3**. The merged datasets displayed optimal precision, recall, F1 scores and accuracy (0.84, 0.85, 0.88, and 0.85, respectively).

**Table 3:** Results obtained by the RF models constructed with different datasets.

Datasets	AUC		Precision	Recall	F1-score	Accuracy
	Micro-average	Macro-average				
T2WI	0.940	0.840	0.790	0.800	0.790	0.800
DWI	0.920	0.840	0.800	0.830	0.800	0.830
ADC	0.920	0.780	0.800	0.810	0.800	0.810
Merge	0.930	0.900	0.840	0.850	0.880	0.850

## 4 Discussion

In this study, radiomic signatures were extracted from MR images to construct ternary ML classification models for predicting the P504s/P63 status, which shed new light on the diagnosis of prostate disease.

To the best of our knowledge, this is the first report to achieve the goal of a noninvasive and accurate PCa diagnosis method *via* an MRI-based ML approach for IHC biomarker prediction. The constructed classifiers exhibited good predictive ability between label 1 (P504s-/P63+: BPH) and label 2 (P504s+/P63-: PCa) states; notable, the RF algorithm performed especially well (label 1: sensitivity: 0.917, specificity: 0.857; label 2: sensitivity: 0.962, specificity: 0.909, respectively). This signifies that our models possess good diagnostic ability for PCa and BPH and decrease the number of required biopsies. Yi-Yan Zhang et al. (21) reported a PSA density (PSAD)-related ML method that enhanced the detection rate of PCa (sensitivity: 0.866; specificity: 0.781), while our P504s/P63-related ML technique further improved upon this diagnostic performance. However, we found that the models showed good specificity and accuracy (0.980, 0.831) but poor sensitivity (0.333) for label 0. The reason for this phenomenon might be as follows. On the one hand, the relatively small sample size of the label 0 group for model training resulted in a lower true-positive rate (i.e., sensitivity). On the other hand, label 0 represented an atypical hyperplasia group, ranging between benign and malignant tumors. Histopathologically, atypical hyperplasia does not exhibit classic heterogeneity, while from the imaging perspective, atypical nodular hyperplasia can also cause restricted water diffusion (22), leading to difficulties in case identification and misdiagnosis.

In our study, 32 meaningful radiomic features were selected: 27 texture features and 5 first-order statistics. Of these 27 prominent texture contributors, 7 were GLCM features and 9 were the GLRLM features. These GLCM and GLRLM features represented gray value changes between pixels and could reflect the complexity and heterogeneity of lesions (23), demonstrating a great ability to differentiate between benign, intermediate, and malignant tumors because the malignant tumors had more inhomogeneous internal structures than the benign and intermediate lesions (24). Moreover, cancer cells have rapid and overcrowded growth patterns as well as an insufficient supply of blood and oxygen, which causes cell hypoxia and the formation of various types of neovasculature. Additionally, different levels of tumor aggressiveness result in different manifestations. All of these characteristics displayed significant spatial differences. Histogram analysis (i.e., first-order radiomics) is an approach that captures the distribution of individual voxel value intensities that may quantify tumor heterogeneity in a routine MRI volume of interest (VOI), and some tumor studies have suggested that such analyses indeed assisted with the evaluation of diagnoses, biologic aggressiveness and prognoses (25–28). However, the performance gains of models built with first-order statistics are relatively limited when the spatial variations between voxels are not considered. Texture parameters maximize the information extracted from medical images to reflect disease relations from cell morphology microheterogeneity and molecular expression to imaging microheterogeneity, this was proven to be reliable in distinguishing malignant from benign tissue and useful for improving the predictive ability of classification techniques in multiple studies (29, 30).

Additionally, this study revealed the strengths of T2WI in biomarker prediction because all highly ranked radiomic features were derived from T2-weighted images; this finding was consistent with that of a prior study (31). Our previous study (32) also found that the entropy derived from T2WI and the kurtosis, skewness, uniformity, and entropy derived from ADC maps showed a good ability to differentiate between high-grade PCa (HGPCa) and non-high-grade PCa (NHGPCa). Similar to our findings, the results of P. Xing et al. (33) also demonstrated that whole-volume histogram and texture analyses of T2WI and ADCs could provide efficient evidence for clinical decision making. However, Bonekamp et al. showed that added value comes from ADCs rather than T2WI (34). Although the underlying causes were unknown, we speculated the following. First, different MRI parameters have different emphases: quantitative ADC maps, resulting from the cell density and mobility of water molecules around lesion changes, are commonly used to estimate the malignancy degrees of tumors and risk-stratify patients. However, P504s/P63 represent the structures and expression product transformations in prostate epithelial cells and basal cells, respectively. Therefore, T2 is more likely to represent structural changes. Second, clearly resolved T2WI images might provide more details about diseases. Moreover, we applied different feature selection methods and procedures, leading to different radiomic features.

For the five final constructed ML predictive models, the RF algorithm (accuracy: 0.85; F1 score: 0.75; recall: 0.74) was superior to the other four algorithms (the GBDT, LR, AdaBoost and KNN). This finding was consistent with that of P. Chiu et al. (35). An RF algorithm (36) builds each tree independently in parallel and integrates the results at the end, contributing to noise reduction and prediction accuracy improvement. Some studies (37) have suggested that an RF algorithm is a stable, popular and efficient decision tree algorithm in practical classification applications. In contrast to our study, Muhammad Arif et al. (36) found that a linear model and the KNN algorithm had better validity. The inconsistencies in the literature may occur under different model application conditions, as further discussed below. LR (38) involves techniques for determining the effects of multiple independent variables on a dependent variable. Logistic sigmoid units are typically used to output (class) binary classifiers rather than ternary classifications, while KNN relies on several nearest neighbor points for classification and is known to work reliably in smaller datasets, as has been shown in previous studies (39). However, KNN may not be suitable for classification in this case since potentially significant impacts may be caused by the k value, the distance calculation and appropriate predictors (40). Finally, ML algorithms are sensitive to the input sample size. The GBDT (41) and AdaBoost (42) both require massive data volumes for training and correction based on feedback. The small sample size in the present study may explain why the GBDT and AdaBoost methods generally achieved moderate performance. Therefore, it seems more reasonable to select specific algorithms for target study situations.

In addition to the influencing factors mentioned above, the background of disease (e.g., inflammation) and race should be taken into consideration. Some patients included in the study concurrent with prostatitis. Although the relationship among prostatitis, BPH and PCa remains inconclusive, some studies indicated that these disorders promote each other. Prostatitis patients were more likely to suffer with BPH, and prostatitis or BPH contributing to a rising risk of PCa (43). In some cases, the effects caused by these changes and consequent treatments (44) were indistinguishable from suspected cancerous lesions at MRI. P504s and P63 indicators couldn’t make a more detailed analysis in prostatitis, either, which could potentially cause confusion to the model. On the other hand, the differences in markers expression of differing racial cohorts might lead to variable results. Hence, our models may not applicable to ethnically diverse populations, as seen in other malignancies (45).

This study had several limitations. First, the final were based on biopsy materials rather than radical prostatectomy, which might have induced a sampling error. Second, P504s+/P63+ and P504s-/P63- were merged into one group (label 0) instead of being discussed separately. Third, the models didn’t discriminate between clinically significant PCa (csPCa) and not-csPCa, the predictions focus on the integrity of the basal membrane and expression of P504s by malignant cells. What’s more, in terms of PCa, the region of interest (ROI) was not subdivided into transitional and peripheral zone lesions. Finally, this study was based on a single-center and small-scale population and some clinical features such as demographic, racial, and biochemical characteristics were not included.

## Conclusion

This radiomic ML study based on MRI images showed that the RF classifier could effectively predict the immunopathologic expression status of P504s/P63 before surgery to achieve the goal of a noninvasive and accurate PCa diagnosis method. However, further research will be necessary to confirm these findings in a larger sample and to determine whether ML models will provide a novel strategy for pathological index prediction and further change clinical decision making regarding the initial prostate biopsies of patients with PCa.

## Data Availability Statement

The raw data supporting the conclusions of this article will be made available by the authors, without undue reservation.

## Ethics Statement

The studies involving human participants were reviewed and approved by The Second Affiliated Hospital of Chongqing Medical University Ethics Review Board. Written informed consent for participation was not required for this study in accordance with the national legislation and the institutional requirements. Written informed consent was not obtained from the individual(s) for the publication of any potentially identifiable images or data included in this article.

## Author Contributions

Conception and design: X-JH, Y-FL, and XS. Collection and assembly of data: Y-FL, XS, X-FQ, and G-YA. Data analysis and interpretation: SQ and Y-FL. Manuscript writing: Y-FL, X-JH, and XS. Final approval of the manuscript: All authors

## Funding

This work was supported by grants from the General Program of Chongqing Natural Science Foundation (cstc2019jcyj-msxmX0073), the Joint Project of Chongqing Health Commission and Science and Technology Bureau (2019GDRC011), the National Major Science and Technology Projects of China (2018AAA0100703), the High-Level Medical Reserved Personnel Training Project of Chongqing and the Kuanren Talents Program of the Second Affiliated Hospital of Chongqing Medical University.

## Conflict of Interest

The authors declare that the research was conducted in the absence of any commercial or financial relationships that could be construed as a potential conflict of interest.

## Publisher’s Note

All claims expressed in this article are solely those of the authors and do not necessarily represent those of their affiliated organizations, or those of the publisher, the editors and the reviewers. Any product that may be evaluated in this article, or claim that may be made by its manufacturer, is not guaranteed or endorsed by the publisher.
